# Association mapping and molecular marker development for cassava brown streak disease resistance

**DOI:** 10.3389/fpls.2026.1977387

**Published:** 2026-10-16

**Authors:** Camilo E. Sánchez-Sarria, Samar Sheat, Stephan Winter, Edward Kanju, Xiaofei Zhang, Nelson Morante, Carmen A. Bolaños, Luz A. Gómez-Martínez, Adriana Bohorquez-Chaux, Edda Mushi, Emmanuel Mrema, Kasele Salum, Ting Yang, Tong Wei, Jonathan Newby, Winnie Gimode

**Affiliations:** 1 International Center for Tropical Agriculture (CIAT), Palmira, Colombia; 2 Plant Virus Department, Leibniz Institute DSMZ-German Collection of Microorganisms and Cell Cultures, Braunschweig, Germany; 3 International Institute for Tropical Agriculture (IITA), Dar es salaam, Tanzania; 4 Department of Plant Sciences, University of California, Davis, Davis, CA, United States; 5 Tanzania Agricultural Research Institute (TARI), Mwanza, Tanzania; 6 State Key Laboratory of Genome and Multi-omics Technologies, BGI Research, Shenzhen, China; 7 International Center for Tropical Agriculture (CIAT) Asia Office, Vientiane, Lao People's Democratic Republic

**Keywords:** cassava, cassava brown streak disease (CBSD), cassava brown streak virus (CBSV), kompetitive allele-specific PCR (KASP), marker-assisted selection (MAS), quantitative trait locus (QTL)

## Abstract

**Introduction:**

Cassava brown streak disease (CBSD) – caused by Cassava brown streak virus (CBSV) and Ugandan cassava brown streak virus (UCBSV) – severely threatens cassava production and food security in sub-Saharan Africa. Breeding for resistance is the most sustainable control strategy, but it requires reliable resistance sources and a clear understanding of the underlying genetic mechanisms.

**Methods:**

In this study, we utilized resistance introduced from Latin American cassava germplasm and mapped the genetic basis of CBSD resistance relevant to African environments. Whole-genome sequencing (WGS) was applied to a segregating population consisting of 426 F1 progeny derived from crosses with CBSD-resistant lines and genome-wide association studies (GWAS) conducted to dissect the genetic basis of resistance.

**Results and discussion:**

A major QTL on chromosome 11 was consistently and significantly associated with CBSD resistance across multiple GWAS models tested and marker types (SNPs and indels), explaining up to 34.89% of the phenotypic variation. Three Kompetitive Allele Specific PCR (KASP) markers were developed and validated from the associated SNPs. All three markers successfully differentiated between known resistant and susceptible parental lines and the mapping population, and one remained significantly associated with resistance in a broader validation population. Interestingly, the same resistance region was previously reported in genetic mapping of East African germplasm, highlighting the central role of this locus across diverse genetic backgrounds. Developing these markers targeting the major chromosome 11 QTL provides a practical molecular tool to accelerate marker-assisted selection and facilitate the development of resistant cassava varieties, ultimately enhancing food security and supporting farmer livelihoods in regions affected by CBSD in Africa.

## 1 Introduction

Cassava (*Manihot esculenta* Crantz) is one of the most important staple crops globally. It serves as a key dietary and economic resource for millions of people across tropical and subtropical regions (Legg et al., 2014; Adebayo, 2023). Global cassava production exceeds 330 million tons, with Africa contributing more than 60% of the total output as it is the continent’s highest-yielding crop per unit area (FAOSTAT, 2023). Its widespread importance is attributed not only to its inherent resilience to abiotic stresses such as drought and low soil fertility but also to its relative tolerance to many pests and diseases, which has allowed it to thrive where other staple crops may fail (Hillocks et al., 2002; Herrera-Campo et al., 2011; Legg et al., 2015). However, recent decades have seen increasing challenges from emerging pests such as whiteflies, which exacerbate the impact of viral diseases including cassava brown streak disease (CBSD), thereby threatening yield stability and food security especially in Africa (Legg et al., 2015; Patil et al., 2015; Chikoti and Tembo, 2022).

CBSD is caused by two viruses: Cassava brown streak virus (CBSV: *Ipomovirus brunusmanihotis*) and Ugandan cassava brown streak virus (UCBSV: *Ipomovirus manihotis*), both members of the genus *Ipomovirus* (Winter et al., 2010; Walker et al., 2022). These viruses cause symptoms on leaves and stems, however, most impact is from the severe necrosis on tuberous roots only visible at harvest (Legg et al., 2014; Chikoti and Tembo, 2022). The viruses are transmitted by whiteflies (*Bemisia tabaci*) (Maruthi et al., 2005, 2017) and the main means of spread is by exchange and use of infected stem cuttings for planting. The severity of CBSD symptoms depends on plant genotype, virus strain, virus titer and environmental conditions (Masumba et al., 2017; Nzuki et al., 2017). CBSD is particularly devastating in East and Central Africa, where root necrosis can render cassava storage roots unmarketable and cause yield losses of up to 70% (Legg et al., 2011; Chikoti and Tembo, 2022). This has profound implications for food security and farmer livelihoods, highlighting the urgent need for effective control strategies (Legg et al., 2014; Nzuki et al., 2017).

Breeding for CBSD resistance offers a sustainable long-term solution (Kawuki et al., 2016; Bizimana et al., 2024), however, this requires a deep understanding of the genetic mechanisms underlying resistance (Nzuki et al., 2017; Wolfe et al., 2017; Nandudu et al., 2023). Historically, breeding programs have utilized resistant parental lines in phenotypic recurrent selection to introgress CBSD resistance into elite varieties, and ongoing efforts integrating new breeding technologies such as genomic selection continue to enhance the efficiency and effectiveness of these approaches (Kawuki et al., 2016; Nzuki et al., 2017; Kayondo et al., 2018; Mukiibi et al., 2019). Despite these methodological advances, progress has long been constrained by the complex infection biology of the causal viruses and the limitations of conventional field phenotyping, which introduce significant uncertainty regarding the actual infection status of plants.

Recently, cassava germplasm with high resistance to CBSD has been identified from Latin American sources (Sheat et al., 2019); however, the genetic basis of this resistance remains unknown. Leveraging these sources has been a challenge because phenotyping for CBSD in the field is time-consuming, taking several years, and often suffers from low accuracy, limiting selection efficiency. Additionally, field phenotyping for CBSD in regions currently beyond the disease frontier such as Latin America and the Caribbean (LAC) and Asia remains a challenge. Genetic mapping efforts, including quantitative trait locus (QTL) mapping (Masumba et al., 2017; Ferguson et al., 2023) and genome-wide association studies (GWAS) (Kawuki et al., 2016; Nzuki et al., 2017; Kayondo et al., 2018; Nandudu et al., 2023, 2024; Ospina et al., 2024) have identified several genomic regions associated with CBSD resistance, yet these discoveries have not been translated into practical molecular markers for marker-assisted selection (MAS).

Building on previous research that addressed the challenges of accurately phenotyping for cassava brown streak virus resistance through controlled screening (Sheat et al., 2019; Sheat and Winter, 2023), we utilized parental lines with established resistance profiles for this study. Prior infection studies identified cassava germplasm from Latin America showing high resistance to the viruses causing CBSD, as well as genotypes with contrasting responses to virus infection. The cassava accession COL 40, when graft-infected with either virus, showed no leaf symptoms, and the absence of virus was confirmed by quantitative reverse transcription PCR (qRT-PCR). Other accessions, such as COL 144, displayed differential resistance, being resistant to CBSV but susceptible to UCBSV. Moreover, another group of genotypes showed no leaf symptoms after inoculation, but root necrosis associated with high virus concentrations was recorded (Sheat et al., 2019, 2022; Sheat and Winter, 2023). Expanding upon these findings, this study aimed to:

- Identify QTL associated with CBSD resistance in a genetically diverse cassava population generated from crosses with parents that were stringently selected for resistance against the viruses, CBSV and UCBSV (Sheat et al., 2019, 2022).
- Convert lead single nucleotide polymorphisms (SNPs) into robust Kompetitive Allele-Specific PCR (KASP) assays applicable in MAS as there are currently no known markers in use for selection of this trait.

Among available genotyping options, KASP was selected because breeding applications targeting a specific locus require a cost-effective, uniplex assay, and is routinely utilized across major cassava breeding programs for targeted MAS due to its high-throughput capability, lower per-sample cost for screening large populations with predefined markers, co-dominant scoring accuracy, and flexibility in assay design for validation. We developed KASP markers linked to the identified loci, providing breeders with tools for the efficient selection of resistant plants. The application of these markers will accelerate the development of resistant cassava varieties, ultimately improving food security and supporting millions of farmers.

## 2 Materials and methods

### 2.1 Mapping populations and phenotyping

A diverse cassava population of 426 accessions was developed at CIAT, IITA and TARI from crosses among parents with complementary traits to combine CBSD and cassava mosaic disease (CMD) resistance (**Supplementary Table S1**). Most of the progeny was from Latin American germplasm, either COL40 (resistant to both CBSV and UCBSV) or COL144 (resistant to CBSV) (Sheat et al., 2019), as well as several other progenitors (**Supplementary Table S1**). Botanical seeds from these crosses were shipped to the Leibniz Institute DSMZ in Germany, transplanted into soil and kept at 38 °C and 70% humidity in a growth chamber for germination for approximately 4 to 6 weeks. Seedlings with 3 to 5 leaves were then transferred to a foil tunnel in the glasshouse. After 4 weeks, two-node cuttings were taken from each seedling and rooted for further propagation (Sheat et al., 2022).

For virus infection and indexing, the workflow described in Sheat et al., 2019 was followed, combining virus inoculation, symptom scoring on leaves and roots, and qRT-PCR-based virus detection to accurately define resistance phenotypes. Virus infections were established in 8-week-old cassava seedlings by side grafting as described by Winter et al. (2010). Two buds from CBSV-Mo83-infected TMS 96/0304 donor plants were grafted onto each of the 2 recipient seedlings, and the graft union was sealed with parafilm for approximately 10 days. Plants were maintained under high humidity immediately after grafting and later transferred to glasshouse conditions. No formal spatial blocking or randomization was applied in the glasshouse layout, which is acknowledged as a limitation of the study design. However, because plants were maintained under tightly controlled glasshouse conditions and conservative genotype-level classification was used the risk of phenotype misclassification was considered minimal. Symptom development on the leaves was assessed four weeks after grafting in which symptomatic plants were classified as susceptible whereas symptomless plants (showing no symptoms on leaves) considered putatively resistant, were subjected to qRT-PCR for virus detection. The appearance of CBSD symptoms on leaves and stems, even in only one seedling, was considered indicative of susceptibility of the respective genotype. Leaf symptoms were visually assessed for at least 5 months and plants that remained asymptomatic were additionally evaluated for root symptoms 9 months after grafting. Crucially, qRT-PCR was employed primarily as a diagnostic assay to confirm virus presence or absence and to support genotype-level phenotype classification. Because tissue sampling was not performed under a standardized design intended for quantitative viral-load tracking across all genotypes, tissues, and time points, cycle threshold (Ct) values were not utilized as a quantitative trait for GWAS or for Ct-versus-symptom correlation analyses.

The symptom scoring introduced by Sheat et al. (2019) was based on a severity scale, defined as: S0, no symptoms/no virus; S−, S+, no virus on the leaf (S-) with moderate symptoms on roots (S+); S+, mild symptoms on leaves only/virus found in leaves; S++, severe symptoms on leaves/virus found in leaves; and S+++, very severe symptoms on leaves/plant death. For mapping purposes, we converted the descriptive severity scores to a 1–5 scale, where: S0 = 1; S−/S+ = 2; S+ = 3; S++ = 4; and S+++ = 5. The accessions and corresponding CBSD scores are summarized in **Supplementary Table S1**.

Samples from leaves, stems, and roots were collected for qRT-PCR to confirm virus presence and to refine the assessment of resistance phenotype and response to infection (Sheat et al., 2019).

### 2.2 Genotyping and marker analysis

Genomic DNA was extracted from freeze-dried young leaf tissue harvested from the 426 seedlings using a modified CTAB method (Doyle, 1991). DNA quality and concentration were assessed using a NanoDrop™ One spectrophotometer, and integrity was verified by electrophoresis on a 1% agarose gel. Subsequently, the samples were sent to BGI Shenzhen for 10x whole-genome sequencing (WGS) on a DIPSEQ platform with 100-bp paired-end reads. Variant calling was performed by aligning the sequences to version 8.1 of the *Manihot esculenta* genome (https://phytozome-next.jgi.doe.gov/info/Mesculenta_v8_1) with bwa mem (version 0.7.12) (Li and Durbin, 2009), and single nucleotide polymorphisms (SNPs) and insertions/deletions (indels) were identified using GATK (version 4.1.8) (DePristo et al., 2011). Two VCF files were therefore obtained, one for SNPs and another for indels. Using vcftools (Danecek et al., 2011), both datasets were filtered based on a threshold of 10% missing data (missingness), 5% minor allele frequency (MAF), quality score > 30 (Q), read depth (DP) per individual genotype, and mean depth (mean-DP) per site ranging between 10 and 40. We applied depth and mean-depth filters to ensure uniform and adequate sequencing coverage across the population, which enhances mapping power in association studies (Nielsen et al., 2011). This approach also improves the accuracy of indel detection by minimizing false positives and false negatives associated with coverage variability (O’Rawe et al., 2013).

Additionally, the linkage disequilibrium (LD, r^2^) decay across the whole genome was determined in a window of 10 kb using PopLDdecay (Zhang et al., 2019). Further, LD pruning of SNPs was done using a 10 kb window size, a step of 50, and an r^2^ threshold of 0.8 using PLINK (Purcell et al., 2007). A non-aggressive LD pruning strategy was applied to avoid removal of informative SNPs (Purcell et al., 2007; Uffelmann et al., 2021). All downstream SNP analyses were performed using the LD-pruned final dataset. No LD pruning was performed on indels, given their lower genomic density, higher genotyping error rates, and reduced statistical robustness compared to SNPs (Li, 2014; Rakocevic et al., 2019). To assess population structure and the clustering of accessions, a principal component analysis (PCA) was performed using the final set of filtered SNPs. The analyses and the plotting were done in R (R Core Team, 2020), using the following packages: SNPRelate (Zheng et al., 2012) for the PCA and ggplot2 for the plotting (Wickham, 2016).

### 2.3 GWAS and candidate gene identification

Genome-wide association analyses were performed for both SNPs and indels using Genomic Association and Prediction Integrated Tool version 3 (GAPIT3; Wang and Zhang, 2021) package in R (R Core Team, 2020). Three different models were employed (i) generalized linear model (GLM), (ii) multiple loci mixed model (MLMM) and (iii) Bayesian information and linkage disequilibrium iteratively nested keyway (BLINK) (Wang and Zhang, 2021). The 1–5 composite CBSD response/severity score was used as the phenotype, with genotype treated as the unit. The two grafted plants per genotype were used to support conservative genotype-level classification rather than as independent entries. To control for population structure, the first three principal components (PCs) were included as fixed-effect covariates (Q-matrix) across all three models. For MLMM, cryptic relatedness was further controlled by incorporating a genomic relationship matrix (K-matrix) as a random effect, calculated via the VanRaden algorithm. BLINK addressed relatedness through a fixed-effect framework by iteratively selecting pseudo-QTNs (covariates in linkage disequilibrium with causal variants) based on the Bayesian information criterion. Significant loci were identified based on a threshold of Bonferroni correction (Bonferroni, 1936) α/m where α is the significance level (in this case, α = 0.05), and m is the number of tested hypotheses (in our case, the number of SNPs and indels analyzed in each GWAS). To evaluate model fit and potential genomic inflation, Quantile-Quantile (Q-Q) plots were generated, and the genomic inflation factor (λ) was calculated for each model. The proportion of phenotypic variance explained (PVE) was estimated for lead markers. Candidate genes were identified within a 20 kb window (10 kb upstream and downstream) of the significant loci. This proximity window was applied as a practical threshold to prioritize immediate positional candidate genes.

### 2.4 KASP marker development and validation

The selection of SNPs for KASP conversion was guided by a multi-stage filtering process to ensure both statistical relevance and practical utility in breeding. We first prioritized lead SNPs on chromosome 11 that exhibited the highest phenotypic variance explained (PVE) and/or consistent significance across different models. Technical feasibility also played a critical role in the final selection, as some SNPs either did not amplify or appeared monomorphic. Markers were converted into KASP assays by Intertek Group plc, Australia, using flanking sequence information and their standard assay and thermal cycling conditions. Three assays met standard quality criteria — clear genotype clustering, >90% call rate, and robustness across DNA concentrations — and were validated in cassava germplasm with well-characterized CBSD phenotypes (Sheat et al., 2022). Since most of these genotypes have been used as progenitors and shared widely globally, we also tested them using the cassava mosaic disease (CMD2) S12_7926132 marker (Rabbi et al., 2022). For further validation of the three CBSD markers, a separate population of 108 individuals from various progenitors were tested. This validation panel was distinct from the population used for the initial marker discovery and included accessions from other pedigrees not represented in the discovery set. Technical validation was assessed via KASP call rates. Additionally, phenotypic validation was performed by constructing a confusion matrix to determine the overall performance metrics of the markers in predicting the phenotypes. True positives (TP) were defined as resistant accessions (scores 1 and 2) correctly identified by the marker. True negatives (TN) were assigned to susceptible accessions (score of ≥3) correctly classified. From these values, the accuracy, sensitivity, specificity, and positive and negative predictive values were calculated using the following formulae (Platten et al., 2019):


$$\text{Accuracy}=\;\frac{\text{TP}+\text{TN}}{\text{TP}+\text{FN}+\text{FP}+\text{TN}}$$

$$\text{Sensitivity}=\;\frac{\text{TP}}{\text{TP}+\text{FN}}$$

$$\text{Specificity}=\;\frac{\text{TN}}{\text{TN}+\text{FP}}$$

$$\text{Precision }(\text{Positive prediction value}:\text{ PPV})=\;\frac{\text{TP}}{\text{TP}+\text{FP}}$$

$$\text{Negative prediction value }(\text{NPV})=\;\frac{\text{TN}}{\text{TN}+\text{FN}}$$

## 3 Results

### 3.1 Phenotypic variation of the mapping population

Of the 426 accessions, 177 (41.5%) were virus-free (score of 1). Among the infected accessions, 26 (6.1%) showed very mild symptoms (severity score of 2), 131 (30.8%) displayed moderate symptoms (score of 3), and 91 (21.4%) displayed severe symptoms (score of 4). Only one accession (0.2%) was severely affected, exhibiting complete wilting (score of 5) (**Supplementary Table S1**). When severity scores were grouped into resistant (scores 1-2) and susceptible (scores ≥ 3), the phenotypic population segregated at an approximate ratio of 1:1. Both COL40 and COL144 progenies were well represented in the mapping population, but they differed slightly in their Resistant: Susceptible (R:S) ratios. COL40 progeny showed an approximate 1:1 ratio, whereas COL144 progeny presented a higher proportion of S accessions, approximating an R:S ratio close to 1:2 (**Figure 1**).

**Figure 1:**
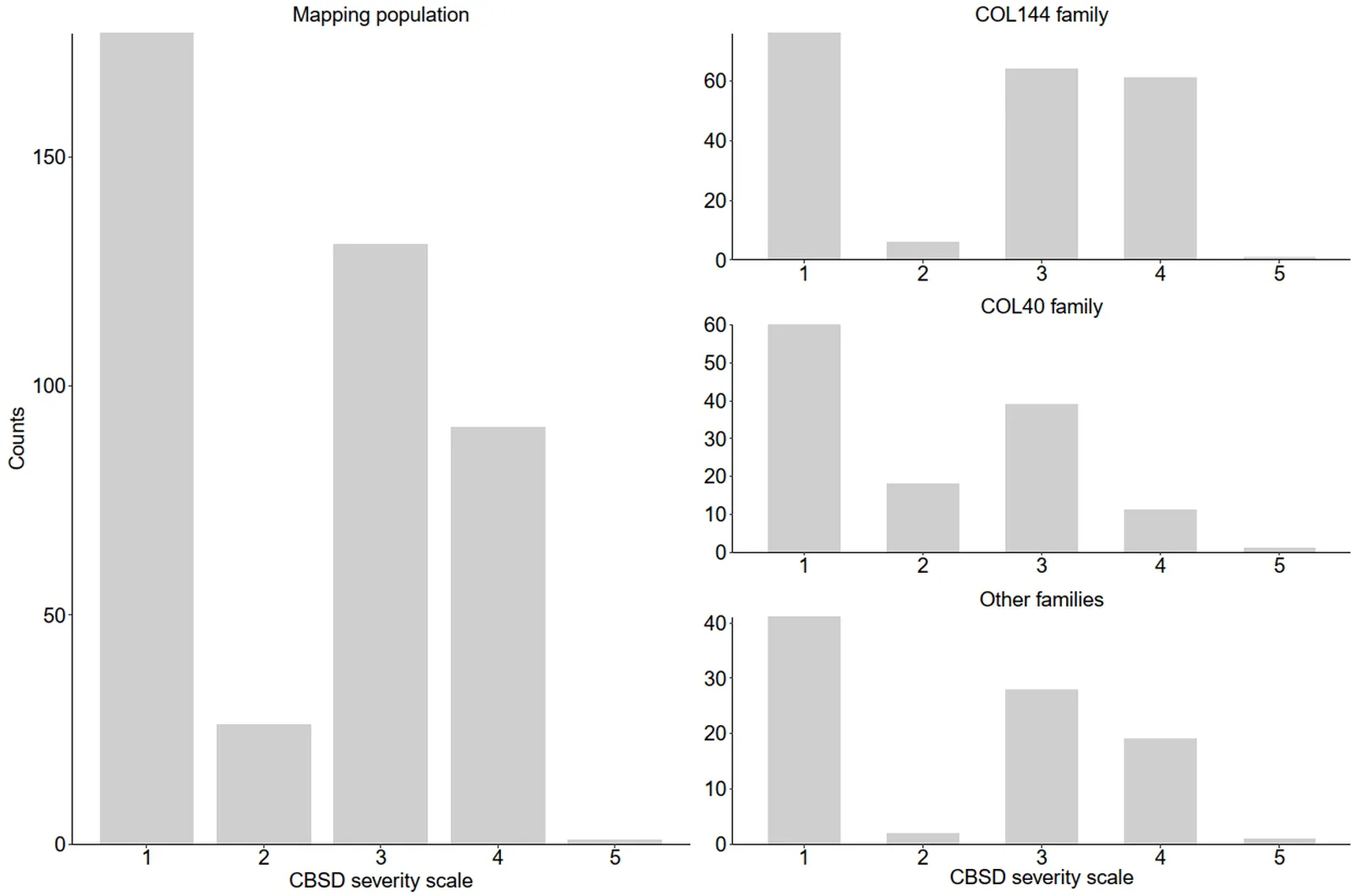
Frequency distribution of cassava brown streak disease (CBSD) severity scores (scale 1–5) for the mapping population (*N* = 426). Data is shown for the entire mapping population and grouped by main pedigree groups: COL144, COL40, and other families.

### 3.2 Genotyping and association mapping

A total of 28,066,278 SNPs and 4,948,914 indels distributed across all 18 cassava chromosomes were obtained following whole-genome sequencing. After all filtering steps, a final dataset of 395,681 SNPs (**Figure 2A**) and 439,710 indels (**Supplementary Figure S1**) were retained and used for association mapping. The LD decay across the whole genome had a maximum LD (r^2^) value of 0.47, decreasing to 0.1 at approximately 500 kb (**Figure 2B**).

**Figure 2:**
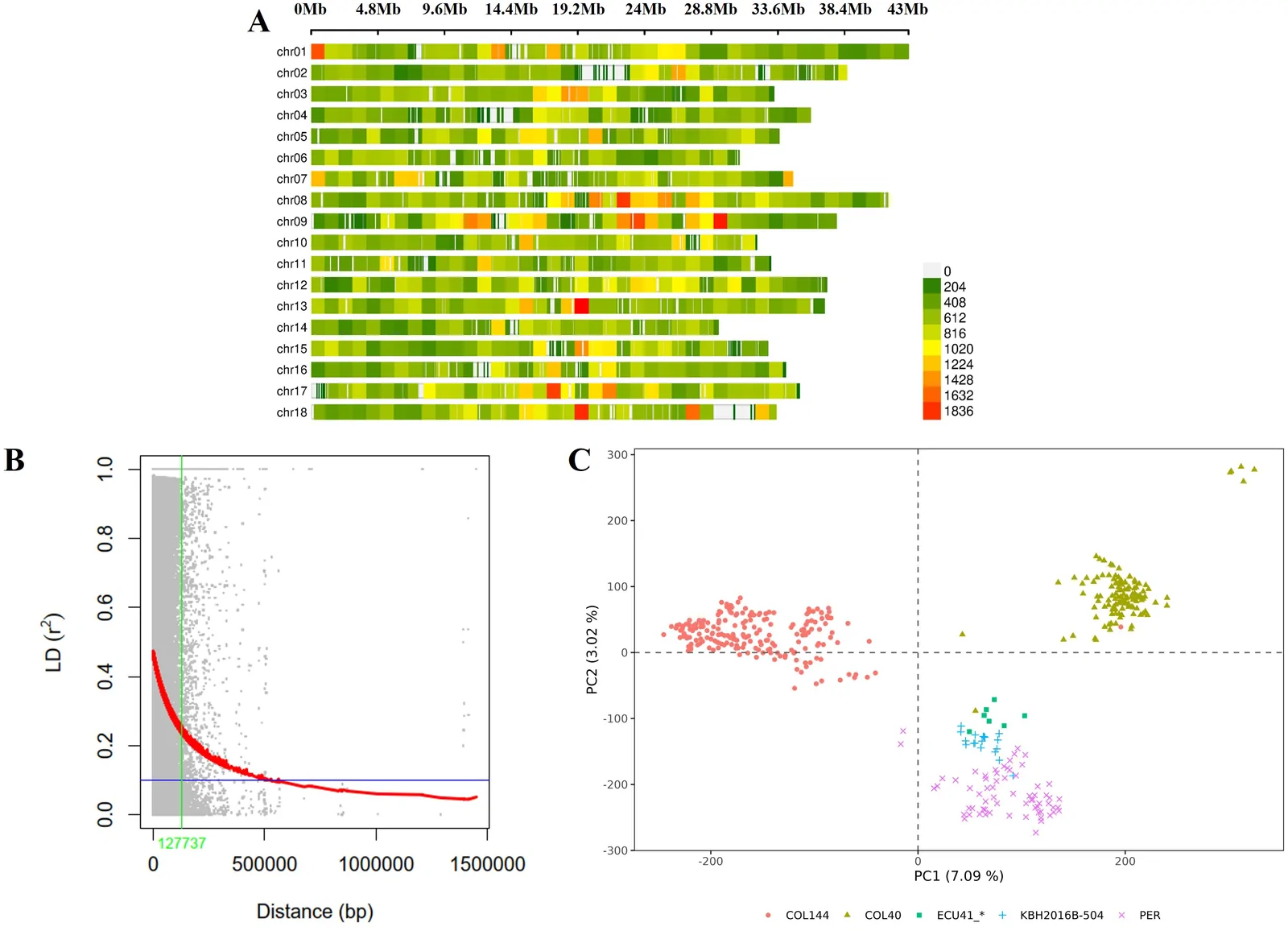
Summary statistics of the SNP dataset: **(A)** marker distribution and density across the 18 chromosomes; **(B)** genome-wide linkage disequilibrium (LD); and **(C)** principal component analysis (PCA) of the 426 genotypes, highlighting parental cross information.

To understand the population structure, we performed a principal component analysis using the 395,681 SNPs. The first two principal components captured 7.09% and 3.02% of the genetic variance. Clear clusters were observed on the PCA plot, with accessions grouping together based on their shared parentage (**Figure 2C**). Specifically, we observed a cluster for genotypes with COL144 genetic background and another for those with COL40 background. A third cluster was formed by accessions with Peruvian pedigrees (PER353 and PER221). Finally, a minor cluster constituted accessions from two donors: Open-pollinated ECU41, and KBH2016B-504. To account for this structure in our research, we included the first three principal components, which collectively explained 12.77% (PC3 = 2.66%) of the observed genetic variation, in our subsequent association analyses. The three PCs were selected based on visual inspection of the eigenvalue scree plot inflection points (**Supplementary Figure S2**).Genome-wide association analyses identified a cluster of significant SNPs on chromosome 11 as the primary genetic signal for CBSD resistance, particularly between positions 27.9 and 30.9 Mb (**Figure 3**; **Table 1**). The most significant SNP in the GLM model was C11_28362507 (A/G) (*p* = 5.18 × 10^-15^; MAF = 0.158), explaining 4.24% of the phenotypic variance. Several additional SNPs in strong linkage disequilibrium within this region—such as C11_28049495, C11_28138538, C11_28179191, and C11_28295117—showed consistent effects and statistical support (**Table 1**).

**Figure 3:**
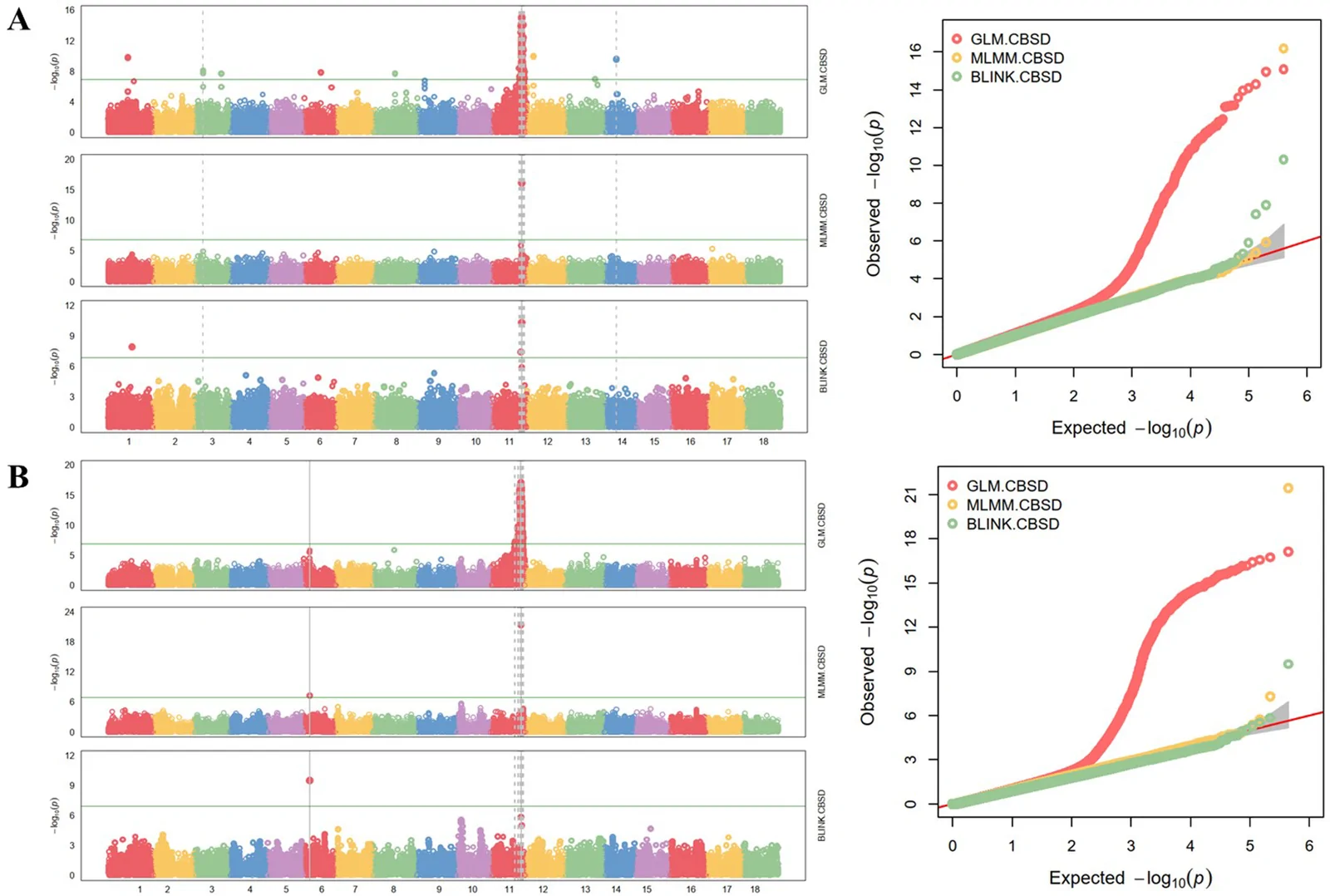
Manhattan and quantile–quantile (QQ) plots from genome-wide association studies (GWAS) of CBSD severity, using three models (GLM, MLMM, and BLINK) applied to SNP **(A)** and indel **(B)** datasets. The green horizontal lines in the Manhattan plots indicate the Bonferroni-corrected significance threshold.

**Table 1:** List of significant markers with a phenotypic variance explained greater than one (PVE > 1) identified through genome-wide association studies (GWAS) based on SNPs and Indels.

Dataset	Model	Marker	Alleles	p value	MAF	Effect	PVE
SNPs	GLM	C03_7944849	C/T	1.64E-08	0.150	-0.758	6.11
		C11_27919279	A/G	1.10E-08	0.218	0.726	1.98
		C11_28049495	T/G	6.44E-12	0.165	-0.926	2.80
		C11_28127112	T/C	8.74E-09	0.122	-0.892	1.38
		C11_28138538	G/A	5.34E-11	0.155	-0.954	1.45
		C11_28179191	A/G	8.18E-13	0.163	-0.977	3.57
		C11_28295117	T/C	2.23E-10	0.217	-0.779	2.07
		C11_28362507	A/G	5.18E-15	0.158	-1.111	4.24
		C11_28586243	C/T	1.05E-08	0.169	-0.769	1.79
		C11_29023596	G/C	9.93E-13	0.256	0.870	2.22
		C11_29190633	G/C	3.04E-09	0.222	-0.734	1.76
		C11_29275084	C/G	8.34E-15	0.147	-1.157	1.33
		C11_30652870	A/G	3.87E-08	0.344	0.715	1.61
		C11_30935285	A/G	1.19E-08	0.278	0.648	1.63
		C14_10446224	A/G	2.29E-10	0.128	-1.043	4.01
	MLMM	C11_28362507	A/G	6.69E-17	0.158	-1.110	33.36
	BLINK	C01_22447810	C/T	1.23E-08	0.276	0.443	5.02
		C11_27608777	G/A	3.84E-08	0.099	0.647	10.22
		C11_28303522	T/C	4.88E-11	0.373	0.779	34.89
Indels	MLMM	C06_6149419	AT/A	4.95E-08	0.248	0.49	7.32
		C11_28682610	GT/G	3.65E-22	0.257	-0.71	17.72
	BLINK	C06_6149419	AT/A	3.21E-10	0.248	0.45	12.58

The MLMM model confirmed the major locus at C11:28362507, which explained 33.36% of the phenotypic variance. Likewise, the BLINK model identified a neighboring SNP, C11_28303522 (T/C), as the top hit with the highest PVE of 34.89% (**Table 1**). These results were further supported by quantile-quantile (QQ) plots demonstrating proper model calibration and effective control of population structure against the expected null distribution. The calculated genomic inflation factors (λ) for the GLM, MLMM, and BLINK models were 1.124, 1.009, and 1.028, respectively, indicating that the multi-locus models provided superior control over spurious associations (**Figure 3**).

Additional association signals were detected on chromosomes 1, 3, 6, 8, 12, 13, and 14 using the GLM model (e.g., C03_7944849, PVE = 6.11%; C14_10446224, PVE = 4.01%), but these signals were not consistently retained in the other models and generally explained lower proportions of the phenotypic variance (**Supplementary Table S2**).

To further validate the stability of our findings, we performed a ‘leave-family-out’ sensitivity analysis by systematically excluding specific families and iteratively rerunning the GWAS. This procedure ensured that the genomic associations were not disproportionately influenced by some crosses with larger representation and that results remained consistent across the broader population (**Supplementary Figure S3**; **Supplementary Table S3**). Additionally, to address the ordinal nature and class imbalance of the 1–5 severity scale, we performed a complementary binary association analysis contrasting symptom-free and virus-free accessions (score of 1) against symptomatic individuals (scores of 2–5). This binary transformation confirmed that our major chromosome 11 resistance associations are robust and not driven by class distribution artifacts in the higher severity tails (**Supplementary Figure S4**).

In the indel-based mapping, the strongest association was also located on chromosome 11 at position 28.6 Mb (GT/G), with a *p*-value of 3.65 × 10^-22^ and PVE of 17.72% (**Table 1**). A second indel on chromosome 6 at 6.1 Mb (AT/A) was significant across both MLMM and BLINK models, explaining up to 12.58% of phenotypic variation (**Table 1**).

### 3.3 Candidate genes

Our candidate gene analysis focused on genes within a 20 kb window (± 10kb) of significant genetic variations (SNPs or indels). We acknowledge, however, that genetic variants may exert effects at greater genomic distances. While we observed some uncharacterized genes near the significant loci, our candidate gene analysis prioritized those with existing functional annotations or domains to facilitate meaningful interpretation (**Table 2**).

**Table 2:** Candidate genes identified based on significant markers with a phenotypic variance explained greater than one (PVE > 1) using the cassava reference genome v8.1.

Marker	Dataset	PVE	Model	Gene ID	Gene start and end position	Annotation	Marker position	Distance to marker (bp)
C03_7944849	SNPs	6.11	GLM	Manes.03G065616	7947959 - 7963787	paramyosin-related	Upstream	3110
C11_28127112	SNPs	1.38	GLM	Manes.11G120900	28128322 - 28132181	crinkly4	Upstream	1210
C11_28138538	SNPs	1.45	GLM	Manes.11G120900	28128322 - 28132181	crinkly4	Downstream	6357
C11_28179191	SNPs	3.57	GLM	Manes.11G121233	28173921 - 28177891	Leucine-rich repeat protein kinase family protein	Downstream	1300
C11_28295117	SNPs	2.07	GLM	Manes.11G121700	28287411 - 28293111	Far-red impaired responsive (FAR1) family protein	Downstream	2006
C11_28295117	SNPs	2.07	GLM	Manes.11G121800	28301083 - 28304943	pfkB-like carbohydrate kinase family protein	Upstream	5966
C11_28362507	SNPs	4.24	GLM	Manes.11G122200	28350697 - 28354708	Leucine-rich repeat protein kinase family protein	Downstream	7799
C11_28362507	SNPs	4.24	GLM	Manes.11G122600	28366944 - 28369376	RHOMBOID-like protein 13	Upstream	4437
C11_28362507	SNPs	4.24	GLM	Manes.11G122700	28370121 - 28373671	Protein of unknown function (DUF1336)	Upstream	7614
C11_29023596	SNPs	2.22	GLM	Manes.11G127300	29016692 - 29024845	Ypt/Rab-GAP domain of gyp1p superfamily protein	Inside	1249
C11_29023596	SNPs	2.22	GLM	Manes.11G127500	29026975 - 29036694	Alkaline-phosphatase-like family protein	Upstream	3379
C11_30652870	SNPs	1.61	GLM	Manes.11G140900	30643319 - 30647486	zinc finger (C3HC4-type RING finger) family protein	Downstream	5384
C11_30652870	SNPs	1.61	GLM	Manes.11G141000	30654867 - 30675470	RNAligase	Upstream	1997
C11_30935285	SNPs	1.63	GLM	Manes.11G144600	30927505 - 30933115	DHBP synthase RibB-like alpha/beta domain	Downstream	2170
C11_30935285	SNPs	1.63	GLM	Manes.11G144700	30933823 - 30941563	4-hydroxy-3-methylbut-2-enyl diphosphate synthase	Inside	1462
C11_30935285	SNPs	1.63	GLM	Manes.11G144800	30943436 - 30944648	hydroxyproline-rich glycoprotein family protein	Upstream	8151
C14_10446224	SNPs	4.01	GLM	Manes.14G130800	10434266 - 10436293	Dynein light chain type 1 family protein	Downstream	9931
C14_10446224	SNPs	4.01	GLM	Manes.14G131100	10451146 - 10455890	(1 of 1) PTHR31134:SF1 - TRANSMEMBRANE PROTEIN 128	Upstream	4922
C11_28362507	SNPs	33.36	MLMM	Manes.11G122200	28350697 - 28354708	Leucine-rich repeat protein kinase family protein	Downstream	7799
C11_28362507	SNPs	33.36	MLMM	Manes.11G122600	28366944 - 28369376	RHOMBOID-like protein 13	Upstream	4437
C11_28362507	SNPs	33.36	MLMM	Manes.11G122700	28370121 - 28373671	Protein of unknown function (DUF1336)	Upstream	7614
C11_28303522	SNPs	34.89	BLINK	Manes.11G121800	28301083 - 28304943	pfkB-like carbohydrate kinase family protein	Inside	1421
C11_28303522	SNPs	34.89	BLINK	Manes.11G121900	28306748 - 28311343	Integral membrane HRF1 family protein	Upstream	3226
C11_28303522	SNPs	34.89	BLINK	Manes.11G122000	28313030 - 28315964	salt tolerance homologue	Upstream	9508
C06_6149419	Indels	7.32	MLMM	Manes.06G027000	6146890 - 6148086	CAP (Cysteine-rich secretory proteins, Antigen 5, and Pathogenesis-related 1 protein) superfamily protein	Downstream	1333
C11_28682610	Indels	17.72	MLMM	Manes.11G125500	28667801 - 28675189	Plant protein of unknown function (DUF936)	Downstream	7421
C11_28682610	Indels	17.72	MLMM	Manes.11G125600	28683290 - 28684655	Phosphoenolpyruvate carboxylase family protein	Upstream	680
C11_28682610	Indels	17.72	MLMM	Manes.11G125700	28685714 - 28689243	Ribosomal L38e protein family	Upstream	3104
C06_6149419	Indels	12.58	BLINK	Manes.06G027000	6146890 - 6148086	CAP (Cysteine-rich secretory proteins, Antigen 5, and Pathogenesis-related 1 protein) superfamily protein	Downstream	1333

For each gene, the gene ID, genomic coordinates (start and end positions), dataset(s), GWAS model(s) in which it was identified, functional annotation, position and physical distance in base pairs (bp) relative to the closest marker, are reported.

From the significant loci found in the GWAS analyses, we obtained a total of 222 genes, with 125 fully annotated (**Supplementary Table S4**). Genes closest to the SNPs and indels explaining the highest variation on chromosome 11 include leucine-rich repeat (LRR) protein kinase family protein (Manes.11G122200), rhomboid-like protein13 (Manes.11G122600), proteins of unknown function (DUF1336: Manes.11G122700 and DUF 936: Manes.11G125500), carbohydrate kinase family protein (Manes.11G121800), HRF1 protein (Manes.11G121900), salt tolerance homologue (Manes.11G122000), phosphoenolpyruvate carboxylase (Manes.11G125600) and ribosomal L38e protein (Manes.11G125700). On chromosome 6, the closest gene to the indel is the CAP (Cysteine-rich secretory proteins, Antigen 5, and Pathogenesis-related 1 protein) gene (Manes.06G027000) (**Table 2**).

### 3.4 KASP marker development and validation

Of the most significant markers selected for KASP assay design, only C11_28303522 (PVE = 34.89) amplified successfully. Other markers either appeared monomorphic or failed to amplify. Consequently, we explored designing KASP primers for other SNPs within the significant region of chromosome 11. We successfully designed KASP assays for two additional SNPs: C11_28313412 and C11_28573659 (**Supplementary Table S5**; **Supplementary Figure S5**). Violin plots of these three markers on the mapping population are shown in **Figure 4**.

**Figure 4:**
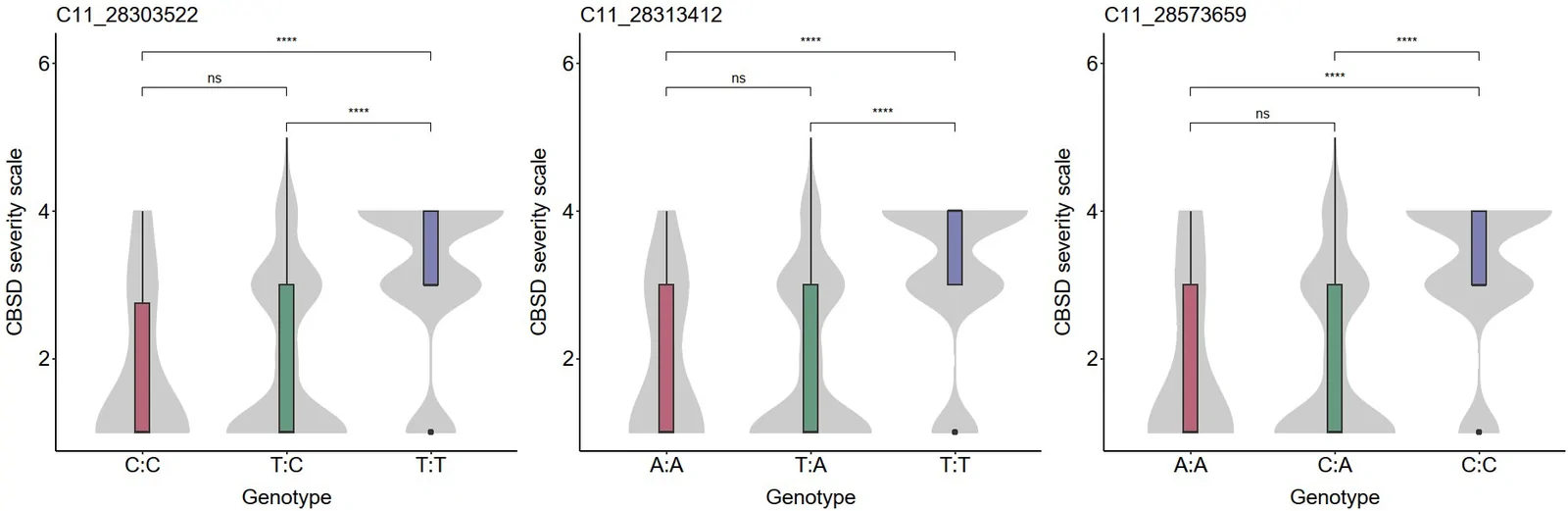
Phenotype–genotype violin plots displaying the performance of three KASP markers for CBSD resistance on the mapping population. The asterisks indicate levels of statistical significance. ****significant at *p* ≤ 0.0001; ns, non-significant (*p* > 0.05).

In the panel of 16 progenitor lines tested by Sheat et al. (2022), all CBSD-susceptible lines shared a similar genetic profile, possessing the unfavorable allele for all three markers (**Table 3**). In contrast, COL40 and PER556, both known to be resistant to both CBSV and UCBSV, had the favorable allele for all three markers. The genotype COL2182, also known for its resistance to both viruses, presented a more complex pattern. It was heterozygous for one marker and had the unfavorable allele for the other two. Among the four genotypes resistant only to CBSV—COL144, PER221, PER333, and ECU41—all were heterozygous for the C11_28573659 marker. PER221 carried the favorable allele for the other two markers, while COL144 and PER333 had the favorable allele for only one other marker. Genotypes where the virus was restricted to the roots generally had the unfavorable allele but were often heterozygous for the C11_28573659 marker (**Table 3**), suggesting a potential partial effect of this marker in limiting viral movement. Analysis of the CMD marker (CMD2 locus) showed that several Latin American accessions were heterozygous, suggesting they may possess field resistance to CMD (**Table 3**).

**Table 3:** KASP marker validation on progenitors.

Accession	C11_28303522	C11_28313412	C11_28573659	CBSD phenotype	Accession	S12_7926132	CMD phenotype
COL40	C:C	A:A	A:A	Resistant to CBSV and UCBSV	COL40	G:G	CMD susceptible
PER556	C:C	A:A	A:A		PER556	G:G	
COL2182	T:T	T:T	A:C		COL2182	G:G	
COL144	C:C	T:T	A:C	Resistant to CBSV	COL144	G:G	
PER221	C:C	A:A	A:C		PER221	G:G	
PER333	C:T	A:A	A:C		PER333	T:G	Potentially CMD resistant based on marker data
ECU41	C:T	T:T	A:C		ECU41	T:G	
PER206	T:T	T:T	A:C	Virus restricted to roots	PER206	T:G	
PER226	T:T	T:T	A:C		PER226	T:G	
PER353	C:T	A:T	A:C		PER353	T:G	
PER597	T:T	T:T	A:C		PER597	T:G	
PER368	C:T	A:A	C:C		PER368	G:G	CMD susceptible
C19	T:T	T:T	C:C	Susceptible to CBSV and UCBSV	C33	T:G	CMD resistant
C33	T:T	T:T	C:C		C413	T:G	
C413	T:T	T:T	C:C		C19	T:G	CMD susceptible
C243	T:T	T:T	C:C		C243	T:G	

The Intertek SNP IDs for the markers used are: C11_28303522 = snpME00590 (favorable allele = C); C11_28313412 = snpME00592 (favorable allele = A); C11_28573659 = snpME00594 (favorable allele = A); S12_7926132 = snpME00021 (favorable allele = T).

In the validation population of 108 individuals, only one of these markers (C11_28573659) remained significantly associated with CBSD, with a significant difference (*p* = 2.22 × 10^-16^) observed between the accessions with the favorable allele and those carrying the unfavorable allele (**Figure 5**). Additionally, a confusion matrix revealed C11_28573659 as the most robust marker on the validation panel, achieving the highest accuracy (74.8%) and sensitivity (95.4%). This marker was highly effective at identifying resistant genotypes while maintaining a low rate of false negatives (missing only 3 resistant plants). Further haplotype analysis across the parentals of the mapping population confirmed that this marker segregates in the Latin American germplasm and specifically within the KBH2016B lineage among the African germplasm (**Supplementary Table S6**). These results highlight that while all three markers differentiate between resistant and susceptible progenitors, their predictive power may vary in diverse populations, with C11_28573659 showing the most consistent association with CBSD resistance (**Supplementary Table S7**).

**Figure 5:**
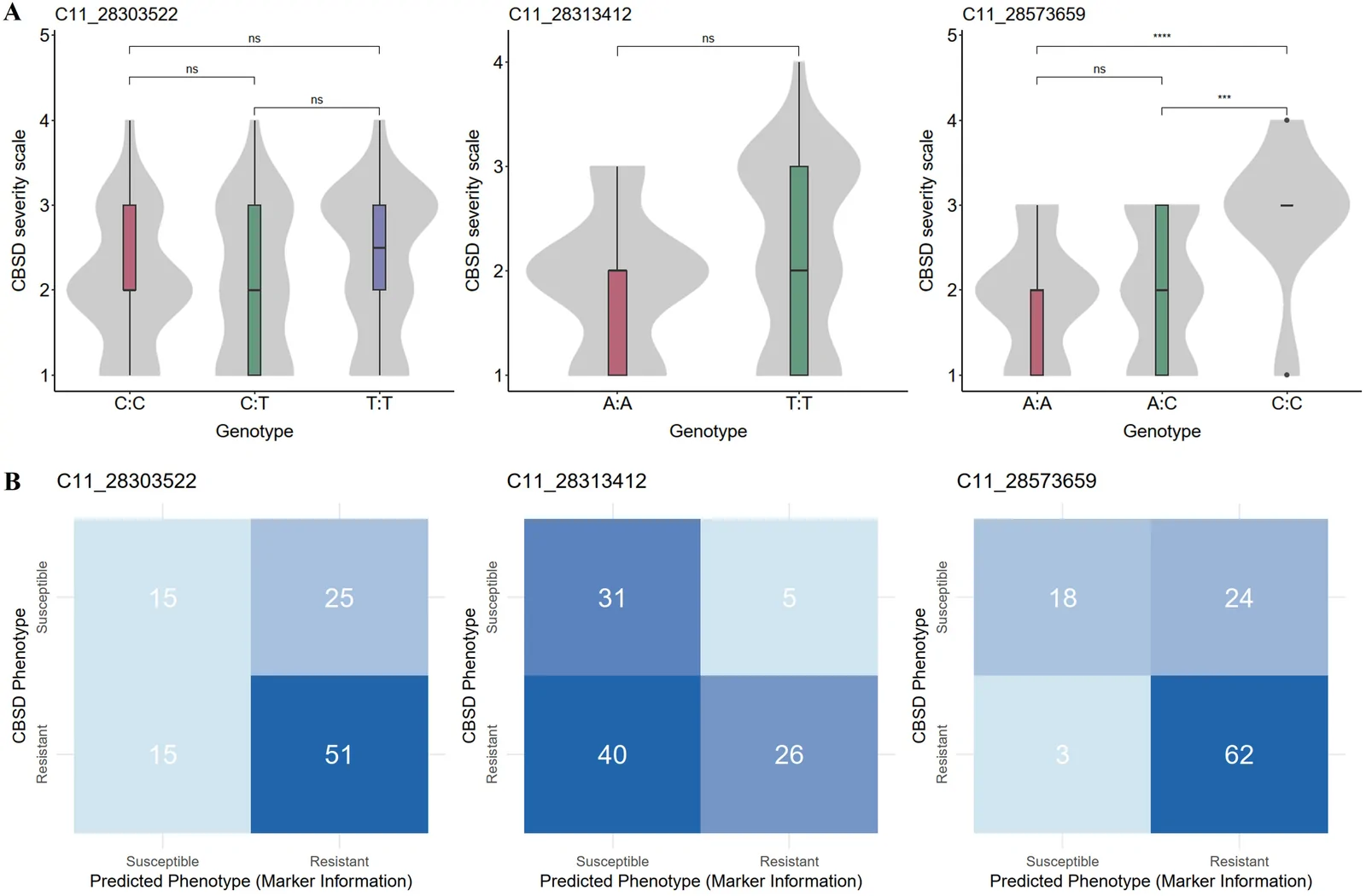
Genetic association and predictive accuracy of three KASP markers for Cassava Brown Streak Disease (CBSD) using a validation panel of 108 cassava genotypes. **(A)** Violin plots illustrating the association between the marker and phenotype. The asterisks indicate levels of statistical significance. ***, **** significant at *p* ≤ 0.001, *p* ≤ 0.0001, respectively; ns, non-significant (*p* > 0.05); **(B)** confusion matrices evaluating the performance of each marker in predicting resistance vs. susceptibility. The matrices compare the observed phenotype against the predicted phenotype derived from marker-specific genotyping.

## 4 Discussion

Our study analyzed seedling populations from crosses with parents clearly defined for their CBSD resistance and resistance screening by artificial infection with known virus isolates (Sheat et al., 2019, 2022). We sought to identify robust QTL associated with CBSD resistance and develop practical molecular markers for MAS. However, because visual symptom scoring alone cannot reliably distinguish the variable responses to CBSV and UCBSV, especially in clones showing differential resistance, qRT-PCR was combined with symptom scoring to provide a more sensitive and accurate phenotyping approach by directly assessing CBSV-Mo83 virus presence. Our phenotypic analysis of the diverse cassava population revealed a wide range of CBSD infection levels, from virus-free to severely affected plants, with an approximate 1:1 resistant-to-susceptible ratio. The differential R:S ratios observed between COL40 and COL144 progeny underscore the varied genetic contributions of different progenitors to CBSD resistance, with COL40 progeny appearing more resistant. Furthermore, complementary binary association analyses contrasting symptom-free and virus-free accessions (score of 1) against symptomatic individuals (scores of 2–5) confirmed that our major chromosome 11 resistance associations are robust and not driven by class distribution artifacts in the higher severity tails (**Supplementary Figure S4**). The principal component analysis effectively revealed the population structure, showing distinct genetic clusters corresponding to the different families. Incorporating these principal components and kinship matrices into our mixed models (MLMM and BLINK) was crucial for controlling false positives (Wang and Zhang, 2021). The robust performance of all models was supported by the well-behaved QQ plots, which showed expected p-value distributions with clear deviations only in the tail regions corresponding to true associations.

A key finding of this study is the consistent and highly significant association of the genomic region on chromosome 11 with CBSD resistance, across all three GWAS models, for both SNPs and indels. This strong and reproducible signal underscores the critical role of this region in CBSD resistance and aligns with previous mapping efforts that have implicated chromosome 11 as a major QTL for CBSD resistance in various cassava populations (Kawuki et al., 2016; Nzuki et al., 2017; Kayondo et al., 2018; Ferguson et al., 2023; Ospina et al., 2024). Kawuki et al. (2016) identified a broad QTL region spanning 19.87 to 23.75 Mb (on genome v6.1) on chromosome 11. This region corresponds to an approximately 24.9 to 28.4 Mb area on the updated genome assembly (v8.1). Subsequent studies have further confirmed the importance of this locus, with Nzuki et al. (2017) identifying a more precise QTL at 21.76–21.87 Mb (v6.1), which falls directly within the larger region identified by Kawuki et al. (2016). Kayondo et al. (2018) and Ospina et al. (2024) also identified markers associated with CBSD resistance at 23.7 Mb and 23.18 Mb, respectively (v6.1), which is the 28 Mb region on v8.1. Ferguson et al. (2023), likewise, confirmed QTL flanking markers in this area, between 28.49 and 28.67 Mb. This consistent finding across different studies and cassava populations is highly significant. The fact that these QTL are associated with either CBSD root necrosis or foliar symptoms, or both, strongly suggests that this genetic locus likely contains multiple closely linked genes, working together or independently to influence resistance to CBSD in various genetic backgrounds. This makes the chromosome 11 locus a high-priority target for cassava breeding programs aiming to develop new, CBSD-resistant varieties. The high PVE by markers in this region (up to 34.89% for SNPs and 17.72% for indels) further highlights its substantial contribution to resistance.

The genes closest to the significant SNPs and indels, particularly on chromosomes 11 and 6, provide compelling candidates for further investigation into CBSD resistance mechanisms. The results suggest that CBSD resistance is a complex, polygenic trait involving multiple biological pathways, including pathogen recognition, signal transduction, and metabolic reprogramming. The presence of a leucine-rich repeat receptor-like kinase (LRR-RLK), Manes.11G122200 and other LRR near the genetic variations (**Supplementary Table S4**), is particularly significant. These LRR-RLKs are well-known for their roles as pattern recognition receptors (PRRs) that detect pathogen-associated molecular patterns (PAMPs) and initiate immune signaling (Soltabayeva et al., 2022; Jiang et al., 2025; Zhiqi et al., 2025). A rhomboid-like protein, Manes.11G1226000, also stands out, as this gene was previously implicated in a CBSD study by Ospina et al. (2024). While the precise function of rhomboid proteins in plant immunity is not fully elucidated, they are known to regulate other membrane proteins (Thompson et al., 2012; Kumar et al., 2021), suggesting a potential role in modulating defense-related protein activity or signaling cascades. The DUF1336 protein, Manes.11G122700, is another intriguing candidate. Described as an enhanced disease resistance 2 protein (EDR2), it is a lipid-binding protein known to be involved in resistance against pathogens like powdery mildew in *Arabidopsis* and grapevine (Tang et al., 2005; Yang et al., 2020). The involvement of EDR2 in pathogenicity and lipid metabolism regulation (Wang and Wang, 2018; Cui et al., 2025) suggests it may contribute to CBSD resistance by altering host-cell membrane integrity or lipid-based signaling pathways. These findings support the hypothesis that multiple pathways possibly contribute to CBSD resistance, including the lignin pathway, as suggested by Ferguson et al. (2023). Additionally, the carbohydrate kinase family protein (Manes.11G121800) is involved in sugar metabolism, which is often reprogrammed during a plant’s response to pathogen attack (Bolton, 2009). The presence of the SNP with the highest phenotypic variation (C11_28303522) within an intron of this gene further highlights the potential importance of metabolic adjustments in the CBSD resistance response. On chromosome 6, the CAP (Cysteine-rich secretory proteins, Antigen 5, and Pathogenesis-related 1 protein) gene might have a role in CBSD resistance as these proteins are well-documented for their roles in plant immunity and pathogen interaction (Tripathy et al., 2021; Jiang et al., 2023; Yuen et al., 2024; He et al., 2025). It is important to note that candidate genes identified near significant associations represent positional candidates based on physical proximity rather than confirmed functional evidence. While local LD extends up to approximately 500 kb, scanning broader genomic intervals requires careful functional prioritization. Consequently, these genes remain hypotheses that warrant further validation through expression profiling, haplotype analysis and functional characterization to confirm their potential roles in CBSD resistance.

Despite identifying highly significant SNPs from GWAS, the initial attempts to convert these top markers into KASP assays proved challenging, with most failing to amplify or appearing monomorphic. This highlights a common practical hurdle in translating GWAS findings into deployable markers in cassava (Esuma et al., 2022). Potential reasons for these failures include the presence of additional polymorphisms in primer binding sites, complex genomic regions, or issues with KASP primer design for specific sequence contexts. To overcome this, in addition to the one successful assay, we strategically selected and developed KASP assays for two other SNPs within the highly significant chromosome 11 region, aiming to increase the number of robust markers.

All three markers showed significant associations in the mapping population. Our validation with progenitor lines (Sheat et al., 2022) showed that the markers distinguished between susceptible lines and known resistant lines like COL40 and PER556, which both possessed the favorable allele. The varied marker results for COL2182 suggest a more complex resistance mechanism in this genotype, possibly involving additional genes or different alleles not captured by these specific markers. All the known susceptible progenitors (Sheat et al., 2022) had the unfavorable allele. In the validation population of 108 individuals, only one of the CBSD markers remained significantly associated with the trait. This may be due to different factors, including a relatively small population size that might have insufficient statistical power to detect associations, genetic background differences, and the polygenic nature of CBSD resistance (Wolfe et al., 2017; Kayondo et al., 2018). The inclusion of both CBSD- and CMD-resistant parents in the mapping population development, and the CMD2 marker in our screening process for the set of progenitors demonstrates a multi-trait breeding strategy, which is essential for developing robust and resilient cassava varieties that can withstand a range of viral threats in the field. Although some genotypes with the dominant, resistant T allele of the CMD2 locus have shown susceptibility in the field (Thuy et al., 2021; Sheat et al., 2022) the identification of South American accessions with this favorable allele is particularly promising and suggests potential for CMD resistance which warrants further field testing (**Table 3**).

While our controlled glasshouse screening successfully established robust associations with the CBSV-Mo83 isolate, we acknowledge that broad resistance across the entire CBSD complex must account for both CBSV and UCBSV strains. The chromosome 11 locus identified here corresponds with major resistance regions previously reported in East African studies where both virus species co-circulate. Nonetheless, future evaluations involving UCBSV challenge and field trials will be critical to confirm whether the marker tools developed here confer universal protection against the broader disease complex. Moreover, root necrosis was not across all the plants and yield were not assessed in this study, the SNP associations reported here relate to overall plant response to virus infection and cannot be interpreted as markers for root necrosis or yield response.

While earlier studies highlighted chromosome 11 as a locus associated with CBSD resistance, this work advances the translation of this region by developing KASP assays linked to the chromosome 11 signal. Here, we confirmed this locus as a major hotspot for CBSD resistance and developed three KASP assays of which one (C11_28573659) successfully retained a significant association in the broader validation population for marker-assisted selection. Their successful development marks a major step toward practical application in breeding. Evaluation of the marker validation parameters (**Supplementary Table S7**; **Figure 5B**) demonstrates that marker C11_28573659 is the most robust candidate for routine deployment. It exhibited high sensitivity (0.954) and a high negative predictive value (NPV; 0.857), indicating that it successfully captures a high proportion of the resistant germplasm. Conversely, its moderate positive predictive value (PPV; 0.721) reflects the presence of some false positives, indicating a limited ability to distinguish all susceptible materials. In a practical breeding context, this implies that while the marker can serve as a useful enrichment or screening tool in specific genetic backgrounds to reduce initial numbers, advanced lines selected as “resistant” by the marker should still undergo some downstream phenotypic evaluation to weed out false escapes. In contrast, marker C11_28313412 exhibited a conservative profile with high specificity (0.861) and PPV (0.839), but a lower sensitivity (0.394), making it more suited for elite parent enrichment rather than population-wide screening. Meanwhile, marker C11_28303522 exhibited an NPV of 0.500, which may render it not as effective for early-stage culling due to the high risk of accidentally discarding true resistance.

In Africa, optimized deployment of C11_28573659 may support product development by accelerating the breeding of CBSD resistant varieties through MAS. Moreover, following further field validation, these tools could support pre-emptive breeding in Asia and LAC, allowing breeders to track resistance alleles without the immediate need for endemic pathogen pressure field screening. The recent spread of cassava mosaic disease (CMD) into mainland Southeast Asia (Uke et al., 2022) and cassava witches’ broom disease (CWBD) into LAC (Pardo et al., 2024) underscores the importance of staying ahead of emerging threats.

Finally, we acknowledge the small sample size limitation. Graft inoculation provided direct and synchronized exposure to the virus, helping to reduce variation associated with field infection, where whitefly access, virus acquisition and transmission efficiency, and infection timing can vary. In addition, evaluating two plants per genotype without formal spatial randomization in the glasshouse also represents a methodological limitation. However, the robustness of our major chromosome 11 QTL across multiple statistical models and sensitivity analyses indicates that this design did not compromise our core genetic findings, though broader multi-environment field validation remains an essential next step.

Given the performance variations observed across the three assays, we exercise caution regarding universal marker readiness. Further efforts should focus on validating these markers in larger, more diverse populations to ensure their broad applicability in global cassava breeding programs. Additionally, a more comprehensive approach could be to explore a broader range of SNPs and indels within the chromosome 11 hotspot to utilize a multi-marker haplotype panel for more precise MAS.

## 5 Conclusion

This study successfully identified a robust and consistent major QTL on chromosome 11 for CBSD resistance in cassava, validated across multiple analytical models and marker types. We also identified several promising candidate genes within this region, providing a foundation for future functional studies. While challenges remain in translating all significant GWAS hits into reliable KASP assays, the validated markers from this locus, and the continued development of additional robust markers from this region, will significantly enhance the efficiency of MAS for CBSD resistance. While independent field-based root necrosis phenotyping remains necessary for validating performance across diverse environments and against both CBSV and UCBSV, these advances will support the development of resistant cassava varieties, improving food security and farmer livelihoods in the CBSD-affected regions.
